# ‘We do not know’: a qualitative study exploring boys perceptions of menstruation in India

**DOI:** 10.1186/s12978-017-0435-x

**Published:** 2017-12-08

**Authors:** Linda Mason, Muthusamy Sivakami, Harshad Thakur, Narendra Kakade, Ashley Beauman, Kelly T. Alexander, Anna Maria van Eijke, Kayla F. Laserson, Mamita B. Thakkar, Penelope A. Phillips-Howard

**Affiliations:** 1 The Liverpool School of Tropical Medicine, Pembroke Place, Liverpool, UK; 2 Tata Institute of Social Sciences, V.N. Purav Marg, Deonar, Mumbai, Maharashtra 400088 India; 3 U.S Centres for Disease Control and Prevention, India Country Office, CDC India, American Embassy, Shantipath, Chanakyapuri, New Delhi 110021 India; 4 Water Sanitation and Hygiene Section United Nations Children’s Fund, New Delhi, India

**Keywords:** Focus group discussions, Menstruation, Perception, Boys, India

## Abstract

**Background:**

In low-middle income countries and other areas of poverty, menstrual hygiene management (MHM) can be problematic for women and girls. Issues include lack of knowledge about menstruation and MHM, and stigma around menstruation, also access to affordable and absorbent materials; privacy to change; adequate washing, cleaning and drying facilities; as well as appropriate and accessible disposal facilities. In order to effect change and tackle these issues, particularly in patriarchal societies, males may need to become advocates for MHM alongside women. However, little is known about their knowledge and attitudes towards menstruation, which may need addressing before they can assist in acting as advocates for change. The present study was undertaken to explore knowledge and attitudes about menstruation among adolescent boys across India, in order to gauge their potential to support their ‘sisters’.

**Methods:**

The study was undertaken across three states in India, chosen a priori to represent the cultural and socio-economic diversity. Qualitative data using focus group discussions with 85 boys aged 13-17 years, from 8 schools, was gathered. Data were analysed using thematic analysis.

**Results:**

The results were organised into three main themes, reflecting the key research questions: boys’ knowledge of menstruation, source of knowledge, and attitudes towards menstruation and menstruating girls. Knowledge comprised three aspects; biological function which were generally poorly understood; cultural rites which were recognized by all; and girls’ behaviour and demeanour, which were noted to be withdrawn. Some boys learnt about puberty and menstruation as part of the curriculum but had concerns this was not in-depth, or was missed out altogether. Most gathered knowledge from informal sources, from overhearing conversations or observing cultural rituals. Few boys openly displayed a negative attitude, although a minority voiced the idea that menstruation is a ‘*disease*’. Boys were mostly sympathetic to their menstruating sisters and wanted to support them.

**Conclusions:**

These findings provide some optimism that males can become advocates in moving forward the MHM agenda. The reasons for this are twofold: boys were keen for knowledge about menstruation, searching information out despite societal norms being for them to remain ignorant, they were also largely sympathetic to their menstruating sisters and fellow classmates and understanding of the issues surrounding the need for good MHM.

## Plain english summary

In some poor regions of the world girls and women have difficulty looking after their menstrual hygiene. They resort to using items such as rags, cloths, foam from a mattress, or leaves and sometimes have nothing at all to soak up the blood. Those who use cloths may struggle to clean and dry them hygienically. When they have access to, and can afford pads, there may be issues with disposal. They are further disadvantaged because they lack knowledge around menstruation and menstrual hygiene management and it may be a taboo subject that they should not talk about. Because this is a gender issue, in societies where women are not empowered girls and women may need the help of boys and men to promote better access to suitable and affordable products, and sanitary support. However, little is known about male knowledge and attitudes towards menstruation, and whether they may be effective champions in helping to resolve menstrual issues alongside women. The present study was undertaken to explore knowledge and attitudes of adolescent boys across India. The study was undertaken across three states in India, chosen to represent a wide cultural and socio-economic range of the population. Qualitative data were gathered using focus group discussions with 85 boys aged 13-17 years, from 8 schools. Data were analysed using thematic analysis. We found there were three main themes: boys’ knowledge of menstruation, how they obtained their knowledge, and their thoughts about menstruation and menstruating girls. Specifically, the boys poorly understood menstruation in terms of the biology; however, they understood some of the cultural restrictions placed on girls during menstruation, and they also knew that menstruation affected girls in a negative way. Some boys learned about puberty and menstruation as part of the school programme, but they felt strongly that they were not given enough information. Most boys obtained their knowledge from listening to women and girls talking, or from watching them. Although a few boys thought that menstruation was a disease, most of them did not think negatively about menstruation, were kind to girls who were menstruating and wanted to support them. From our data, we are hopeful that boys and men can become involved in this gender issue, and help support menstrual issues for girls and women in the future.

## Background

Menstrual hygiene management (MHM) remains an issue of gender inequality [1], particularly for women and girls living in low- and middle- income countries (LMIC) who have limited information and few resources available for their basic menstrual hygiene needs. These include the need to access affordable and absorbent materials, privacy to change, adequate washing, cleaning and drying facilities, and appropriate and accessible disposal facilities. Menstruation is considered to be a contributing factor toward school drop-out, and exacerbates the struggles girls have in attending school and being able to function and participate fully [2, 3]. As a consequence, girls fall behind boys in their education, which prevents them from reaching their potential. An overview from the first ‘MHM in Ten’ global panel meeting reported that for girls in LMIC, lack of access to good MHM has some effect on school outcomes, with ‘repercussions for sexual, reproductive, and general health and well-being throughout the life course’ [4]*.* An agenda setting out future priority action areas for MHM was proposed to address the challenges facing schoolgirls (and women) in LMIC. This also recognised the need to involve men, and groups working to improve male involvement as stakeholders in these activities [5].

In countries such as India where the present study is set, cultural and societal taboos surrounding menstruation place restrictive practices upon menstruating females, compounding the difficulties faced by girls and widening the gender disparity further. Such taboos include restrictions in their diet, participation in cooking and visiting places of worship or involvement with social activities, as well as having to sleep or sit separately from the rest of the household [3, 6, 7]*.* In some settings, menstruating girls and women are considered unclean and untouchable during menstruation [2]. Furthermore, menstrual blood is taboo and so simple tasks such as washing and drying menstrual cloths becomes shameful for girls and women. This may result in damp cloths being stored or worn before adequately dry [3], which is potentially harmful to their health [8]. Even within families, menstruation is exclusively a female issue with males remaining ignorant of health and hygiene matters pertaining to their wives, mothers and children.

MHM is seen as a matter for women only, and one that should be kept private and not discussed, as to do so may bring shame. As a result, girls and women cannot express their needs and affect change within their homes, community or in society in general. It is suggested that male attitudes are one of the main factors driving the stigmatization and myths surrounding menstruation [9, 10]. Recognition and inclusion of the gendered nature of women’s reproductive health is weak, with low priority given to issues of gender equality and positive sexual and reproductive health outcomes resulting in MHM being a neglected issue as noted by Mahon et al. [1]. In the present study context lack of, or weak management responses for good menstrual health, coupled with negative social and gender norms surrounding menstruation and women’s sexual and reproductive health in general has hindered progress in reducing gender inequality within India. India, a patriarchal society has seen its ranking in the Gender Gap Index widen from 98th in 2006 when the Index was created, to 108th in 2015 [11], thus indicating little progress, if any at all in reducing inequality. In a patriarchal society, men hold power over women at all levels of society, women are traditionally seen as second class citizens with little power individually or collectively in relation to politics and finance, as well as health and education [12]. India has been described as a country of ‘contrasts’ with huge disparities of wealth, as well as gender inequity. A recent study reported that just 23% of women took part in family decision making in Rajasthan, compared to twice that (49%) in Tamil Nadu [13]. With little power in decision-making within the family setting, women will have minimal if any, impact on community or societal matters. Low literacy, lack of confidence and cultural norms prevent women from being innovators and it is therefore even more important that males assist in advocacy to affect change. There is a role for men to become involved in menstrual issues to help reduce the restrictive practices and negative view of menstruation, including in Indian and other societies, and to promote better menstrual hygiene management and critical investment in WASH (Water, Sanitation and Hygiene), through their roles as family members, policy makers, stakeholders and investors.

To affect such change, it is necessary to understand the knowledge and attitudes that males currently hold so that appropriate actions can be taken to garner their support as advocates for women, or to contribute of their own accord in addressing these issues. A literature search suggests that male views on menstruation are largely unknown and under-researched globally. A small body of work undertaken in western culture, predominantly cited in feminist literature up to the 1990’s, suggests male perceptions of the menstruating woman are negative [14, 15]. However, little is known currently, nor within LMIC, where women are more likely to suffer disadvantage through lack of MHM. One study of male pupils in Taiwan found menstruation was taboo for boys despite their desire for information [16]. They found both families and teachers refused to talk about it or answer questions, going so far as to forbid boys to discuss the topic. As a result boys developed misconceptions, such as girls menstruated on a weekly basis, as well as negative attitudes. Further studies are necessary to elucidate male views and their understanding of menstruation, to facilitate development of educational packages to address this void [4, 5]. The present study aimed therefore to assess the knowledge and views of school-going boys across India on menstruation. The research questions were: What do boys understand about menstruation? Where have they obtained any knowledge about menstruation? What are their views on menstruation and menstruating girls?

## Methods

### Context

The present study was part of a wider project conducted by a multinational research team to assist UNICEF India’s support of the Government of India, to develop evidence-based guidelines for MHM in schools throughout India. Prior to fieldwork, the project undertook a systematic review of literature published throughout India to map the nationwide status of adolescent MHM and WinS (WASH in Schools) [3]. Fieldwork was then undertaken to understand the experiences, best practice, barriers and challenges of MHM and WASH faced by schoolgirls, and to use the knowledge generated to support a package on MHM guidance for government schools with potential for scale-up across India.

Field-work for the wider project was undertaken across three purposefully selected states representing differing socio-demographic, cultural and geographic profiles across India. These were: Maharashtra, Tamil Nadu and Chhattisgarh. One district was randomly selected from each state. Multi-level stratified sampling was then used to select participants from 53 schools. These included 43 regular government schools, and 10 ‘Model’ schools, representing best practice with strong WASH/MHM activities supported through external agencies, selected by UNICEF. Qualitative data were collected through a series of focus group discussions (FGDs) held with schoolgirls, teachers, parents and schoolboys, in a random sample of these schools. Quantitative data gathered in parallel were collected, with findings reported to UNICEF. The present paper reports on the findings from the FGDs conducted among schoolboys.

### Procedure

Eight FGDs were conducted among a total of 85 boys aged 13-17 years, in three FGDs each in Tamil Nadu (one of which was a ‘model’ school - Tam S21), Chhattisgarh and two FGDs in Maharashtra. Participants were invited by the field research team, after teachers had identified boys who were able to adequately articulate and whose parents consented, and who themselves had assented to join the study. The FGDs were facilitated by trained moderators and a note-taker, both male and female and of Indian nationality. The note-taker assisted with the capture of the main points, group dynamics and non-verbal gestures. The FGDs were conducted in the local language of the state and were recorded. The FGDs began discussing ground rules including the importance of confidentiality. Participants were assigned a number, and stated this as they spoke. This was in order that the recording and subsequent transcript could attribute the quotes to the contributor. Each FGD lasted approximately 30 min and was transcribed verbatim, then translated directly into English and counterchecked using back translation. The transcripts were reviewed for accuracy by the moderator and note-taker and entered into the NVIVO computer package (QSR International), to facilitate analysis and allow transparency between the coding and analysis work.

### Analysis

Thematic analysis was used to reflect the participants’ findings. The transcripts were each reviewed by four members of the multinational team to familiarize themselves with the data. Each of the members then devised a set of codes that were felt to represent the narratives. These codes were merged into an agreed coding frame, and the main themes emerging from the coding frame were described. One of the authors then reviewed each of the transcripts in turn and coded the transcripts using the framework, with additional codes added to the framework as any emerged. A second member of the review team repeated the process for consistency, reaching agreement on the majority of codes and the overall framework. A narrative was woven by placing the coded materials within the appropriate theme, checking that it fit the assigned theme and comparing and contrasting the descriptions to provide clarity on strength of feeling, contradictions and level of agreement. Quotes are presented, with participant codes, for example P1; where participant number is unknown Px is used.

## Results

The results were organised into three main themes to reflect on the key research questions: boys’ knowledge of menstruation, their source of knowledge and their attitudes towards menstruation and menstruating girls. Within these main themes, the following subthemes emerged: denial, misconceptions, curriculum gap, cues to acquire knowledge, loss of friendship / changing relationships, societal views, menstruation as a normal function, disease and isolation, and supporting girls. Interestingly, there were no differences in findings between the model school and the usual schools, however there were some differences in knowledge due to differences in the teaching of menstruation across the 3 states, which is indicated below where relevant.

Following introductions and warm-up, all focus group discussions began with a question exploring whether participants had heard of menstruation and what they understood it to be. The majority of boys denied all knowledge. For a minority this appeared to be true, although for many others it stemmed from reluctance to talk or embarrassment on this subject. This was evidenced across the groups by silence or self-conscious laughter when the moderator tried to initiate discussion. The following is an excerpt demonstrating boys’ reluctance to admit any knowledge:

‘Now, have you heard something called periods or menses? (M).

‘No’ (P2)

‘Have you not heard of that’? (M).

‘No’ (P4).

‘Do you not know anything about that’? (M).

‘We do not know’ (P6).

‘You told that you know’? (M).

‘I said no’ (P8).

‘You told no. okay. Do you know what menses is’? (M).

We do not know’ (P3) (All Tam S6**).**

Despite these strenuous denials, it emerged later in the discussion that these participants were taught about puberty and menstruation at school; with one pupil stating ‘*It came as a lesson in the curriculum’* (P3 Tam S6).

### Extent of knowledge

Boys’ knowledge, however rudimentary, became evident during the course of the discussions. Their knowledge comprised three different aspects; biological function, cultural rites and girls’ behaviour and appearance. Knowledge around the biological function of menstruation varied considerably across, and to some extent within the groups, regarding what boys comprehended about menstruation, ranging from just a vague perception that it involved blood, to full understanding of the reproductive cycle:

‘*Bleeding occur, don’t know anything else’* (Px Chhat S1).

‘*We only have heard that word, but don’t know the reason*’ (Px Mah S9).

Frequently, their knowledge and understanding was commonly based on misconceptions as illustrated by the following:

‘*Menstruation is sort of disease in which blood come out mouth. Girls feel giddiness and fall down anywhere*’ (Px Chhat S11).

‘*It comes once in one month. If their voice change, it is told that they have attended puberty. I think that it comes once in 15 days’* (Px Tam S21).

Boys in just one of the groups displayed a little more accurate knowledge and they reported being taught about it ‘*in 8th standard’.*

‘*Eggs are formed to women till 50s but for men, sperms are formed till death*’ (P3 Tam S2).

All boys appeared to be aware of various customs and taboos that restricted girls’ lives, even if they were not sure why such customs were adhered to, or that they were due to menstruation per se. Descriptions mostly included aspects of seclusion, the forbidding of worship, as well as not being allowed to touch people, animals or food. The following excerpt is typical of the knowledge displayed by participants.

‘*They should not sit near us. They should not touch. We should not drink the same water which they drank. Their food we should not eat. They should not come to temple’* (Px Tam S2).

The third way in which boys demonstrated knowledge of menstruation was in their observations of girls in terms of their absence from school, their changed physical appearance and different demeanour during menses. The boys comments were negative, suggesting that they perceived girls suffered both physically and psychologically at this time. Girls were commonly described as getting ‘*irritated’* or ‘*angry’*, and complaining of pain. Boys perceived girls were also unable to concentrate on studies and isolated themselves from friends and classmates.

‘*She sits separately, which is indicative for menstruation’* (P3 Mah S3).

*‘She looks lazy. Like she is suffering with f*ever’ (Px Chhat S11).

Some participants rather sadly described how girls’ behaviour towards them changed once they reached ‘maturity’ or ‘puberty’, suggesting the boys felt they had lost girls valued friendships.

‘*Yes. They do not talk to us. They do not come near us. They do not ask us anything* (P2 *Tam* S2).

‘*She behaves different. She doesn’t come to close to us. If anyone goes close to her, she denies them’* (P11 Mah S3).

A couple of participants explained the reason for this behaviour was grounded in societal attitudes that frown on girls and boys associating with each other when they reached puberty.

‘*Our opinion changes within ourselves that they have attended the puberty and if I talk to her, people think wrongly about us. So we distance ourselves’* (Px Tam S2).

### Source of knowledge

Boys from four FGDs reported that they were taught about puberty and menstruation in school as part of the curriculum during 8th standard. This included the three schools in Tamil Nadu, where they were taught alongside the girls, one school in Chhattisgarh, but none of the schools in Maharashtra. However, few boys appeared to learn much from the curriculum or other school-based fora. This resulted in some complaints that there were not enough details provided and it was taught ‘*lightly*’. In the two FGDs conducted in Maharashtra boys reported that although part of the syllabus, puberty and menstruation were missed out altogether.

*‘We did not hear in the school. We have one chapter in book, but we are not taught about it. ‘Life cycle’ is the name of chapter’* (Px Mah S3).

It was quite common for boys across all groups to ask the FGD moderator to tell them more about menstruation, as they were eager to learn so that they understood menstruation properly. This also included schools where it was taught within the curriculum. Thus the following question was typical:

‘*Can to tell us how menstruation occurs*?’ (Px_Chhat S11).

The general consensus was that both boys and girls should be given information about menstruation in school, with just a few individuals stating it was unnecessary. Although some boys stated a preference for being taught separately from the girls, others thought both gender should be taught together.

‘*It is better to teach together. Then only, we can become aware of that’* (Px).

*It is a bad thing. It should be taught separately. It is not required for boys’* (Px) (both Tam S21).

The little knowledge that boys had gleaned about menstruation appeared to be primarily from informal sources. Boys said they learned about menstruation mostly from picking up cues through observation of female behaviour, as the girls adhered to customs such as not going to the temple, or staying in seclusion. The other manner in which boys acquired knowledge was from overhearing private conversations between women or girls. Sometimes an event such as a girl having stains on her clothing from leakage at school, or being allowed to go home during school hours, also signalled menstruation. Occasionally boys said they were told something directly by their peers or family.

‘*We heard it from women. But whenever we go near from them, they stop talking about it*’ (Px Mah S3).

*‘Yes, They immediately leave for home after taking permission from sir. They clean the floor and then go. In this way boys come to know*’ (Px_Mah S9).

In a couple of FGDs boys considered this topic was not necessary for boys to know about. More commonly however, boys were of the opinion that menstruation should be discussed more openly in their society because it is a ‘*normal*’ thing.

### Attitudes towards menstruation and girls who were menstruating

Few boys openly displayed a negative attitude, although a minority voiced the idea that menstruation is a ‘*disease*’ and believed it was right that girls should be isolated at this time.

‘*I think menses spread dirt, uncleanness’* (P1 Chhat S11).

*‘Transmission of MC [menstrual cycle, sic]….. As malaria spreads, so there is a chance it may also spread like it’* (Px Chhat S3).

‘*What do you think about them staying outside the house for 3 days?’* (Mod).

*‘We think it is good’* (Px) (Both Tam S2).

However, other boys disagreed with the seclusion and felt that girls should remain with their family at this time to be supported by them.

‘*Sir, whatever is happening, is wrong in this regard because girls need their parents during their menstruation’* (Px).

*‘Why do they need them?’* (Mod).

*‘Because she feels weakness. As she sleeps outside the home, any family member should stay with her there’* (Px) (All Mah S9).

Boys across the FGDs mostly appeared keen to help their ‘sisters’ with their menstruation, some demonstrating a protective bond towards them. Many believed if girls could be open about their status it would be beneficial, by allowing them to share their problems and receive help if they should need it.

‘*They can share, then only we will also come to know. Then only we can take them to hospital, take care of them and we can help them’* (Px Tam S2).

‘*Girls should tell their problems without shying’. ‘Whatever is possible from our side, we will give that. It is not out of our capacity’* (Px Mah S9).

Removing the culture of secrecy surrounding menstruation was also suggested as a way forward to help change societal attitudes, making things easier for menstruating girls. Being ignorant about menstruation was deemed ‘*harmful’.*

‘*Even if government does something, our orthodox traditions will not change. But it will change atmosphere in schools’* (Px Mah S9).

The attitude of wanting to help appeared to stem from boys’ perceptions that menstruating girls suffer, from pain, weakness, and isolation. This was observed in a number of the FGDs.

‘*They do not play with boys. They sit quietly. They do not come out. They look sad*’ (Px Tam S21).

One FGD probed further on whether boys teased girls who were menstruating, with boys strongly denying this.

‘*It happens to all girls so they should not be teased. Nobody does this, nobody teases about this’* (Px Chhat S3).

Some boys identified issues girls were facing when they menstruate at school, and a couple of boys were critical of male teachers who they considered did not help support girls when they menstruate. One solution proffered by a few individuals was that schools should employ more female teachers who girls could more easily talk to.

*‘They feel shy while talking to male teachers. So female teacher should be available in our school’* (Px Mah S3).

*‘If anyone has serious problem then teachers don’t give much attention’* (P2).

*‘ So how can we solve this problem?’* (Mod).

*‘There should be one female teacher and doctor in school’* (Px).

*‘ Girls can’t allow to work during menses. Senior teacher don’t give leave immediately even after students get serious’* (Px) (All Chhat S11).

## Discussion

Currently, there is a dearth of literature exploring male knowledge, and perspectives on menstrual hygiene mostly come secondhand through narratives of women and girls [17–19]. Even fewer studies have specifically explored MHM with males [4, 20, 21]. The current study adds to this small body of knowledge using primary data collected from adolescent schoolboys. Importantly, while there are study limitations that prevent the drawing of strong conclusions from the present study, the findings provide some optimism that males can become advocates in moving forward the MHM agenda. The reasons for this are twofold: boys were keen for more knowledge about menstruation, and were searching for information despite societal norms preferring them to remain ignorant. They were also largely sympathetic to their menstruating sisters and fellow classmates and understood the issues surrounding the need for good MHM. The findings, to some extent, echo the small body of literature to date, with some differences from previous studies, which are discussed in more detail below.

Although knowledge and understanding varied across the different discussion groups, we found that it was a challenge for most boys to understand menstruation because of the surrounding secrecy and the limited information they had access to, much of which was inaccurate, or was pieced together erroneously. Oftentimes they were discouraged to discuss menstruation at all. This has also been reported in studies conducted with adolescents in Taiwan and Rwanda [20–22], who similarly reported lack of knowledge on the link between menstruation and ovulation, and reported beliefs such as menstruation being a punishment from the gods, resulting from injury, or that it is a mental or physical ‘disease’. A consequence of this ignorance and secrecy is the maintenance of the stigma surrounding menstruation and menstrual issues. Ramifications include the improbability that they will become advocates for women in MHM issues as males and fathers, that they will fail to understand how menstruation and hygiene affects their wives and daughters and impacts on the community and society at large. Girls would thus continue to suffer the consequences of cultural and societal norms that impact on their health and education.

Boys in our study were mostly curious about menstruation, evidenced not just by their request for it to be taught in the curriculum but also by the questions they asked our moderators. In another Indian study based in West Bengal, boys also requested menstruation to be included in the school curriculum [23]*.* Chang and colleagues (2013) reported that boys in Taiwan resorted to going online to investigate this topic [20], as did some of our participants. We wonder therefore, why boys are poorly informed when they could be provided with information that they request, which would serve for their future role as husbands, partners and fathers.

Provision of education within the school curriculum to boys in their formative years, when they are eager to learn, and where the content can be managed, would appear to be key. The present study raises concern on the apparent lack of education that boys received. Although part of the curriculum via the Adolescence Education Programme (MHRD 2017), it was seemingly ignored or skipped over by teachers. This phenomena has been reported in studies from Kenya, Bolivia and the Philippines [18, 24, 25], which have similarly described teachers’ discomfort in teaching ‘sensitive’ topics, or who feel ill- equipped to adequately cover topics due to a lack of knowledge [26]. Most often teachers do not possess any formal training or guidelines to teach such sensitive information, and may need specific capacity building to correctly impart information. This is particularly important in coeducational schools where girls and boys are present in the same class and when gender of the students and teachers differ.

Studies also suggest that health information and education in schools may be best taught by those trained specifically for that purpose [27], rather than being covered within the curriculum by class teachers. Other authors contend that school (or general) nurses may be particularly effective in providing sex and relationship education in schools [28].

It was interesting that boys in one of the FGDs strenuously denied that girls were teased, a finding contradictory to a large body of international literature suggesting that girls suffer humiliation from being teased. Although most of this evidence has been collected from girls’ narratives [17–19, 29] boys’ studies have also confirmed this [21]. However similar to our study, in Rwanda Penakalapati (2009) also mentioned instances where boys themselves prevented the girls being teased and assisted them in hiding staining [21]. We have no way of knowing whether the participants in our group were genuine in their denial, or concerned about ramifications if admitting to this. Importantly though, evidence from Ghana suggests that taking part in an MHM initiative, which included an educative element, had a positive effect in boys no longer teasing girls [30]. Given the effects of being teased on girls’ confidence and school attendance, the importance of including boys in MHM initiatives cannot be stressed enough.

In our study it was also noted that boys appear to recognize but are saddened by, the resulting change in the nature of their friendships with girls once they reach menarche. In effect they lose friendships. Chang and colleagues (2012) [16] and Penakalapati (2009) [21] similarly reported loss of friendship was remarked on by their participants, and this is further described by Allen and colleagues studying undergraduates in the US (2011) as a ‘gender wedge’ [31]. This phenomenon may stem from the girls’ own change in attitude on reaching puberty and withdrawing from boys generally, but particularly during menstruation. However, it is also likely to be perpetuated in societies where male/female relationships are restricted, as well as in cultures where girls reaching menarche are seen as ready for marriage. Other authors refer to this as the ‘sexualising of menstruation’ [16].

We acknowledge some limitations in the present study including the disadvantage of using FGDs as the key method of soliciting opinion. Because of peer pressure, individuals may not have felt comfortable in voicing opposing views to the group norm. Whilst some contradictory points of view emerged, mostly narratives showed a high level of agreement, which may indicate acquiescence. However, as there were similarities across the FGDs we are confident that our findings represent an overview of the boys’ perspectives even if not every individual view.

We only included boys who attended non-residential schools. It is possible that boys attending residential schools have a different, possibly greater understanding, of menstruation depending on the curriculum teaching. On the other hand, boys who have dropped out of school, and those who have not attended school were not represented in our study. It is likely that their knowledge and understanding was even poorer than those boys attending school routinely.

We are also aware that the series of FGDs were short in length. We believe that this reflects the lack of knowledge boys had about the subject, which coupled with the sensitivity of the topic, meant that they were not verbose in their responses to the moderator, nor stimulated to converse as a group easily. However, boys did appear to give their perspectives and opinions freely, providing useful insights allowing us to answer the research questions posed.

## Conclusion

We conclude that in order to help drive forward the changes needed in MHM, the male perspective should not be ignored or forgotten. Boys need to be educated and involved in MHM whilst at school, something they appear to desire for themselves. MHM should be included as a critical component of comprehensive sexuality and life skills education, not just as a paper exercise but provided to each and every boy so that they grow up informed. Consideration of the education role needs to be taken seriously, with training provided to those teachers, counselors or school nurses responsible for passing on knowledge to the current and future generations. There is need to promote inclusive, learner-centered, participatory and gender transformative teaching and learning on menstruation, which is based on core principles of human rights and gender equality. Rather than a stand-alone education, menstrual teaching should be considered as an important component of comprehensive education on life skills, development and sexuality. Without such education to challenge social norms, negative perceptions of menstruation will remain, and the desired change will not be effected. However, there is hope that with inclusive education boys can become ambassadors in promoting MHM requirements, particularly in cultures where women and girls do not yet have a voice to do this.

## Abbreviations

FGDs: Focus group discussions
LMIC: Low and middle income countries
MHM: Menstrual hygiene management
WASH: Water, sanitation and hygiene
WinS: WASH in schools
